# Tacrolimus Bridging and Combination Therapy Following Infliximab and JAK Inhibitor Failure in Ulcerative Colitis: A Case Series

**DOI:** 10.1002/jgh3.70443

**Published:** 2026-08-16

**Authors:** Nikil Vootukuru, Patrick Hilley, Darren Wong, Ashish Srinivasan, Peter De Cruz, Matthew Choy

**Affiliations:** ^1^ Department of Gastroenterology & Hepatology Austin Health Melbourne Australia; ^2^ The University of Melbourne, Austin Academic Centre Melbourne Australia

## Abstract

**Background:**

Calcineurin inhibitors such as tacrolimus are established rescue therapies for steroid‐refractory ulcerative colitis (UC). However, their efficacy and safety as bridging or combination therapy following failure of both anti‐tumor necrosis factor (anti‐TNF) and Janus kinase (JAK) inhibitor therapy remain uncertain.

**Methods:**

We conducted a retrospective, single‐center case series at a tertiary referral center between January 2020 and December 2025. Adult patients with treatment‐refractory UC who received tacrolimus after documented failure of infliximab and a JAK inhibitor were included. Demographic, disease, treatment, and outcome data were collected.

**Results:**

Seven patients were included (median age, 37.1 years; median disease duration, 6 years), of whom three presented with acute severe UC. Tacrolimus was used as bridging therapy to ustekinumab (*n* = 4), ozanimod (*n* = 1), or upadacitinib (*n* = 1), and as combination therapy with upadacitinib (*n* = 1). Median tacrolimus treatment duration was 3 months, and median follow‐up was 31 months. Six of seven patients achieved a clinical response, including four who achieved clinical remission. Three patients ultimately required colectomy. No serious adverse events were observed; one patient developed tremor and one experienced mild COVID‐19 infection.

**Conclusion:**

Tacrolimus may be a useful bridging or combination therapy in carefully selected patients with UC refractory to both infliximab and JAK inhibitors. Nevertheless, the substantial colectomy rate underscores the severity of disease in this population and highlights the importance of close monitoring and shared decision‐making. Larger prospective studies are needed to better define the optimal role, duration, and safety of tacrolimus in this treatment setting.

## 1 Introduction

The therapeutic landscape of ulcerative colitis (UC) has evolved considerably since the addition of Janus Kinase (JAK) inhibition to existing advanced therapies. In particular, JAK inhibition has enabled sequential treatment strategies in patients with refractory disease both in the acute and chronic settings [1, 2]. While sequential treatment with JAK inhibition may be effective in biological‐experienced patients, there remains a subset of patients who are refractory to both biologics and JAK inhibition.

The role of calcineurin inhibitors, with cyclosporin and tacrolimus, which exert their immunosuppressive effect by inhibiting T‐cell activation and proliferation [3], is well established in the management of refractory UC [4, 5, 6]. As early as 2003, Högenauer et al. demonstrated that oral tacrolimus was an effective therapy in steroid refractory patients with moderate to severe UC [7] and subsequent randomized controlled trials have confirmed its efficacy [4, 8]. Over the last decade, tacrolimus has also been recognized as an effective therapeutic option following failure of anti‐TNF therapy in IBD [9, 10].

Given concerns of long‐term toxicity, calcineurin inhibitors have traditionally been used as bridging therapies to immunomodulators or biological agents in refractory UC; however, the efficacy and safety of calcineurin inhibitors as sequential, bridging and combination therapy in the JAK era have yet to be defined. We present a case series evaluating the efficacy and safety of tacrolimus as a bridging and combination therapy following inadequate response to both infliximab and JAK inhibitors in patients with UC.

## 2 Methods

A retrospective, single‐center case series was conducted at a tertiary metropolitan referral center. All adult outpatients and inpatients with treatment‐refractory UC who received tacrolimus bridging or combination therapy after documented failure of infliximab and JAK‐inhibitors from January 2020 to December 2025 were identified via the electronic medical record and included in the study.

Bridging therapy was defined as short‐term tacrolimus use to achieve disease control prior to transition to another long‐term therapy, whereas combination therapy referred to the concurrent use of tacrolimus with another advanced therapy to achieve disease control. Therapy failure was classified as either primary nonresponse (PNR), defined as failure to demonstrate improvement in clinical signs or symptoms during induction therapy, or secondary loss of response (SLOR), defined as loss of response after an initial clinical benefit.

Baseline data regarding patient demographics, disease characteristics, and previous IBD therapies and duration of therapy were collected. The follow‐up period was defined as time of last follow‐up. Tacrolimus levels, target maintenance therapy, duration of tacrolimus use, and outcome at time of follow‐up were also recorded.

Tacrolimus was commenced using a standard protocol at 0.05 mg/kg twice daily. Dosage was titrated with a general target trough level of 10–15 mcg/L for the first 4–6 weeks and then 5–10 mcg/L thereafter. Tacrolimus levels were assessed after 48 h for inpatients and after 3–10 days for outpatients with dose adjustment. Tacrolimus target levels and monitoring frequency were determined by the treating gastroenterologist according to disease severity and therapeutic response.

Baseline disease activity and severity were assessed using the full Mayo score. Clinical remission at last follow‐up was defined as a partial Mayo score < 2. Biochemical data including C‐reactive protein (CRP) and fecal calprotectin and endoscopic disease activity (Mayo endoscopic subscore), where available, were recorded at baseline, and approximately 3 months following tacrolimus commencement.

## 3 Results

Seven patients with treatment‐refractory UC who received tacrolimus therapy following documented failure of infliximab and JAK‐Inhibitors were included in this series. One met the criterion for mild UC with Mayo 2 disease on endoscopy, two met the criteria for moderate UC, one met the criteria for severe UC, and three for acute severe UC. The median age at the time of tacrolimus commencement was 37.1 years, with a median UC duration of 6 years. Four patients had left‐sided colitis and three had extensive disease (Table 1). Endoscopic disease severity was classified as Mayo 3 in three patients and Mayo 2 in four (Table 2). Five were managed as an inpatient and two as an outpatient. All patients had previously received infliximab (median duration 7 months) that was discontinued due to PNR (*n* = 4) or SLOR (*n* = 3). Five patients received tofacitinib (median duration 5 months), and two patients received upadacitinib. All but one patient discontinued JAK inhibitors due to either PNR (*n* = 3) or SLOR (*n* = 3). One patient continued upadacitinib therapy following SLOR (Table 1).

**TABLE 1:** Baseline characteristics of patients receiving tacrolimus for refractory UC.

Case	Age (years)	Sex	Clinical				Infliximab			JAK inhibitor				Tacrolimus therapy		
				Montreal location	IBD duration (years)	Past IBD therapy	Dosing	Duration (months)	Failure reason	Type	Dosing	Duration (months)	Failure reason	Tac drug level target (ng/mL)	Target maintenance therapy	Other concurrent IBD treatment
1	19.2	M	IP severe	E3	0.7	Mercaptopurine Mesalamine	10 mg/kg induction + re‐induction	4.2	PNR	Tofacitinib	10 mg BD	4	PNR	10–15	Ozanimod	Prednisolone weaning
2	73.2	F	IP ASUC	E2	8	Vedolizumab Mesalamine Infliximab Azathioprine	5 mg/kg	3	PNR	Tofacitinib	10 mg BD	5	PNR	10–15 for 1 month 5–8 after 1 month	Ustekinumab	Vancomycin 125 mg QID for CDI Prednisolone 5 mg daily
3	37.1	M	OP moderate	E2	11	Mercaptopurine	5 mg/kg 6 weekly	8	PNR	Tofacitinib	10 mg BD	6	SLOR	10–15	Ustekinumab	Pentasa 4 g/daily
4	69.9	M	IP moderate	E3	5.3	Mercaptopurine Tacrolimus suppositories	5 m/kg Q6‐8W	6	SLOR	Tofacitinib	10 mg BD	7	SLOR	10–15	Ustekinumab	Prednisolone weaning
5	18.6	F	IP ASUC	E2	6	Azathioprine	5 mg/kg Q8W	8	SLOR	Tofacitinib	10 mg TDS	0.5	PNR	10–15	N/A—colectomy	N/A
6	36.5	M	IP ASUC	E2	7.6	Azathioprine Mesalazine	5 mg/kg 8 weekly	12	SLOR	Upadacitinib	45 mg daily induction 30 mg daily maintenance	13	SLOR	10–15 for 1 month 5‐10 mg after 1 month	Ustekinumab	Prednisolone weaning
7	46.9	M	OP mild	E3	5.2	Mesalazine Vedolizumab Ustekinumab	5 mg/kg 8 weekly —> 10 mg/kg 8 weekly	7	PNR	Upadacitinib	45 mg induction —1 month	23 (ongoing)	SLOR	6–8	Upadacitinib monotherapy	N/A

Abbreviations: BD—twice a day, IP—inpatient, OP—outpatient, PNR—primary nonresponse, SLOR—secondary loss of response, TDS—three times a day.

**TABLE 2:** Outcomes of refractory UC patients receiving tacrolimus therapy including clinical, biochemical, and endoscopic outcomes.

Case	Baseline			Outcomes after 3 months/prior to tacrolimus cessation				Tacrolimus					Outcomes		
	CRP	FCP	Endoscopy	CRP	FCP	Endoscopy	Clinical	Total tacrolimus duration (months)	Adverse events	Remains on	Target level achieved	Starting dose	Surgery	Therapy at last follow‐up	Follow‐up duration (months)
1	7.7	657	Mayo 3	25.8	989	Mayo 3	Active	3	Nil	No	Yes (4 days)	2 mg BD	Subtotal colectomy + end ileostomy → completion proctectomy	Nil	31
2	9.0		Mayo 3	6.6	186	Mayo 1	Remission	26	Yes —COVID	No	Yes (10 days)	2.5 mg BD	No	Upadacitinib	43
3	11.4	938	Mayo 2	6.7		N/A	Active	0.7	Nil	No	Yes (3 days)	4 mg BD	No	Upadacitinib	64
4	12.6	4140	Mayo 2	0.8	N/A	N/A	Remission on steroids	3	Yes —tremor	No	Yes (4 days)	4 mg BD	Proctocolectomy and Ileopouch anal anastomosis	Nil	39
5	12.8	580	Mayo 3	23.5	N/A	N/A	Active	3 days	Nil	No	N/A	2 mg BD	Proctocolectomy and Ileopouch anal anastomosis	Antibiotics for pouchitis	30
6	2.1	453	Mayo 2	0.4	53		Remission	4	Nil	Yes	Yes (6 days)	5 mg BD	No	Ustekinumab	8
7	0.7	440	Mayo 2	13	14	Mayo 1	Remission	8 (Ongoing)	Nil	Yes	Yes (10 days)	3 mg BD	No	Tacrolimus + upadacitinib	8

After shared decision‐making involving the patient, gastroenterologist, and colorectal surgeon, tacrolimus was initiated with close monitoring as a bridge to ustekinumab (*n* = 4), ozanimod (*n* = 1), upadacitinib (*n* = 1), and as combination therapy with upadacitinib (*n* = 1). Corticosteroid weaning co‐therapy was utilized in four patients. In six patients, the initial target trough level was 10–15 ng/mL, with one patient targeting 6–8 ng/mL in view of concurrent upadacitinib combination therapy.

Tacrolimus duration and treatment strategy varied according to clinical response, patient preference, tolerability, and availability of subsequent advanced therapies. In several patients, tacrolimus therapy was continued due to sustained response in the setting of multiple prior advanced therapy failures and limited remaining therapeutic options, resulting in prolonged tacrolimus‐based combination therapy in select cases. The median duration of tacrolimus therapy was 3 months, and the median duration of total follow‐up time was 31 months. Six patients received trimethoprim‐sulfamethoxazole as *Pneumocystis jirovecii* prophylaxis.

Six patients responded clinically to tacrolimus rescue therapy, reaching the prespecified target trough levels within 10 days, with one primary nonresponder who proceeded to colectomy after 3 days. Four patients achieved clinical remission with tacrolimus‐based therapy (Table 2). One patient achieved clinical remission with concurrent corticosteroids, tacrolimus, and ustekinumab, but was considered at high risk of recurrence and subsequently underwent elective colectomy. Another patient received combination tacrolimus and ustekinumab therapy for 25.5 months before transitioning to upadacitinib monotherapy for the subsequent 17 months following SLOR to combination therapy. One patient received tacrolimus for 4 months with concurrent ustekinumab for 3 months and remained in remission on ustekinumab monotherapy 4 months after tacrolimus cessation. One patient remains on combination therapy with tacrolimus and upadacitinib at 8 months follow‐up.

Four patients remained in steroid‐free clinical remission at last follow‐up with the remaining two patients experiencing treatment failure, ultimately requiring colectomy after 4 and 3 months, respectively. No significant adverse events, in particular renal or hypertensive side effects, were associated with tacrolimus use—one patient developed tremors associated with tacrolimus and one patient developed mild COVID infection.

## 4 Discussion

This case series highlights that tacrolimus therapy may facilitate therapeutic bridging to alternative advanced therapies in a select cohort of patients with treatment‐refractory UC.

Tacrolimus has consistently been shown to be a highly effective therapeutic agent in steroid refractory UC in the short to medium term [7, 11, 12, 13, 14, 15, 16]. Furthermore, in acute severe UC, the use of tacrolimus salvage therapy following failure of intravenous corticosteroid therapy has been shown to be as effective as infliximab salvage [17]. Among the patients in this study, tacrolimus was selected due to its relatively rapid onset of action in the context of severe or highly refractory disease, particularly where there was concern that direct transition to another advanced therapy alone may not provide sufficiently rapid disease control. This was particularly relevant in the outpatient setting, where tacrolimus was utilized to provide short‐term disease control during transition to slower‐onset maintenance therapies.

The optimal therapeutic range for tacrolimus in the early management of refractory UC was established by Ogata et al. as 10–15 ng/mL for the first 2 weeks of therapy [8] and ongoing trough levels of 5–10 ng/mL [18].

The use of calcineurin inhibitors is associated with adverse effects including nephrotoxicity, tremor, and infection [19]. These adverse effects often contribute to clinician hesitancy in initiating tacrolimus therapy. In our study, two patients experienced adverse effects—one with tremor and another with mild COVID‐19; however, there were no serious adverse effects noted. These findings suggest tacrolimus was generally well tolerated in this small cohort. The limited sample size and observational design preclude broader conclusions regarding safety; however, these findings align with the wider literature regarding the safety of tacrolimus in carefully monitored IBD populations [4, 12, 15, 20, 21, 22].

Despite this, the clinical use of tacrolimus in this context has largely been limited by the growing armamentarium of advanced therapies for UC. Since 2017, JAK‐inhibitors have emerged as effective therapies in the management of UC [23, 24] and thus there has been a steady increase in the utilization of these agents in clinical practice. The role of JAK‐inhibitors in UC is expanding with a growing body of literature supporting their role in acute severe UC [25] though there is little evidence regarding the use of tacrolimus following JAK failure.

To our knowledge, this is the only case‐series to date to evaluate tacrolimus therapy following failure of JAK inhibition and infliximab. While this study is hypothesis‐generating and limited by its small sample size, these findings provide preliminary guidance suggesting tacrolimus may represent a bridging strategy and combination therapy in carefully selected patients with highly treatment‐refractory disease.

Importantly, despite initial clinical response, three of seven patients ultimately underwent colectomy. This finding underscores the severe and highly treatment‐refractory nature of this cohort and suggests tacrolimus may delay rather than prevent colectomy in some patients. The risk of nonresponse to tacrolimus therapy in this treatment‐experienced patient population is highlighted by a multicenter retrospective cohort study by Oshima et al. which reported lower rates of clinical remission at 12 weeks with tacrolimus in patients with previous or concurrent use of TNF‐α [26]. Therefore, careful shared decision‐making involving the patient and multidisciplinary team is essential to not only identify patients who may respond to tacrolimus therapy but also patients who are unlikely to deteriorate significantly prior to colectomy in the event of nonresponse.

The main limitations of this study are its small sample size and observational design. Consequently, further prospective studies with large sample sizes are required to establish the role of tacrolimus in this clinical context including long‐term safety, optimal treatment duration and therapeutic target.

## 5 Conclusion

In this cohort of treatment‐refractory UC, tacrolimus facilitated therapeutic bridging or combination therapy with alternative advanced agents in select patients. However, the high colectomy rate underscores the need for careful patient selection, shared decision‐making, and close monitoring. Further prospective studies are warranted to define the optimal positioning, duration, and safety profile of tacrolimus treatment, particularly following the failure of multiple advanced therapies.

## Funding

The authors have nothing to report.

## Ethics Statement

This study was conducted as a retrospective case series using de‐identified patient data. In accordance with institutional policies, formal Human Research Ethics Committee approval was not required for this type of study.

## Conflicts of Interest

D.W. has received speaking fees from Fresenius Kabi; A.S. has served as a speaker for Arrotex Pharmaceuticals and received research support and advisory fees from AstraZeneca, AbbVie, Takeda Pharmaceuticals, and DrFalk Pharma; P.D.C. is supported by an NHMRC Emerging Leader 2 Fellowship and has received research support from AbbVie, Ferring, Shire, Janssen, Pfizer, and Takeda. The other authors declare no conflicts of interest.

## Data Availability

The data that support the findings of this study are available from the corresponding author upon reasonable request.
